# Antibacterial activity of a lectin-like *Burkholderia cenocepacia* protein

**DOI:** 10.1002/mbo3.95

**Published:** 2013-06-05

**Authors:** Maarten G K Ghequire, Evelien Canck, Pierre Wattiau, Iris Winge, Remy Loris, Tom Coenye, René Mot

**Affiliations:** 1 Centre of Microbial and Plant Genetics, University of LeuvenKasteelpark Arenberg 20 box 2460, 3001, Heverlee-Leuven, Belgium; 2 Laboratory of Pharmaceutical Microbiology, Ghent University9000, Ghent, Belgium; 3 Department of Bacteriology and Immunology, Veterinary and Agrochemical Research Centre1180, Brussels, Belgium; 4 Molecular Recognition UnitDepartment of Structural Biology, Vlaams Instituut voor Biotechnologie1050, Brussels, Belgium; 5 Structural Biology BrusselsDepartment of Biotechnology (DBIT), Vrije Universiteit Brussel1050, Brussels, Belgium

**Keywords:** Antagonism, *Burkholderia cepacia* complex, lectin-like bacteriocin, LlpA, MMBL family, planktonic, sessile cells

## Abstract

Bacteriocins of the LlpA family have previously been characterized in the γ-proteobacteria *Pseudomonas* and *Xanthomonas*. These proteins are composed of two MMBL (monocot mannose-binding lectin) domains, a module predominantly and abundantly found in lectins from monocot plants. Genes encoding four different types of LlpA-like proteins were identified in genomes from strains belonging to the *Burkholderia cepacia* complex (Bcc) and the *Burkholderia pseudomallei* group. A selected recombinant LlpA-like protein from the human isolate *Burkholderia cenocepacia* AU1054 displayed narrow-spectrum genus-specific antibacterial activity, thus representing the first functionally characterized bacteriocin within this β-proteobacterial genus. Strain-specific killing was confined to other members of the Bcc, with mostly *Burkholderia ambifaria* strains being susceptible. In addition to killing planktonic cells, this bacteriocin also acted as an antibiofilm agent.

Bacteriocins mediate highly selective antagonism among closely related bacteria but such antimicrobial proteins have not yet been reported in *Burkholderia*. We identified a lectin-like protein of the LlpA family in a *Burkholderia cenocepacia* human isolate that strain-specifically and selectively kills planktonic and biofilm cells of other *Burkholderia cepacia* complex members.

## Introduction

While some members of the β-proteobacterial genus *Burkholderia* exhibit attractive properties for biodegradation of environmental pollutants or growth promotion of plants (Suárez-Moreno et al. 2012), several species represent a threat to animal and human health. The *Burkholderia pseudomallei* group includes the causative agents of human melioidosis, *B. pseudomallei*, and of animal glanders, *Burkholderia mallei* (Galyov et al. 2010). The *Burkholderia cepacia* complex (Bcc), encompassing 17 species, is home to opportunistic pathogens, such as *Burkholderia multivorans* and *Burkholderia cenocepacia*, that cause respiratory infections in cystic fibrosis patients and immunocompromised individuals (Sousa et al. 2011; Vial et al. 2011; Suárez-Moreno et al. 2012). Bcc bacteria are difficult to combat due to high intrinsic antibiotic and biocide resistance, biofilm-forming behavior, and prevalence of multidrug-resistant strains (Horsley and Jones 2012).

A possible strategy to devise alternative anti-*Burkholderia* strategies is to exploit the antibacterial activity of molecules involved in competition among *Burkholderia* strains and the potentially novel molecular targets involved (Chandler et al. 2012). Production of the polyketide enacyloxins by *Burkholderia ambifaria* AMMD enables inhibition of some other Bcc species (*Burkholderia dolosa*,* B. multivorans*) (Mahenthiralingam et al. 2011). Certain Bcc species (*Burkholderia ubonensis*,* Burkholderia vietnamiensis*) are susceptible to capistruin, a lasso peptide ribosomally synthesized by *Burkholderia thailandensis* E264 (a member of the *B. pseudomallei* group) that most strongly inhibits the plant-associated *Burkholderia caledonica* (Knappe et al. 2008). Recently, contact-dependent inhibition systems mediating competition among *Burkholderia* strains were characterized in *B. pseudomallei* and *B. thailandensis* (Anderson et al. 2012; Nikolakakis et al. 2012). The role of bacteriocin-mediated antagonism among cystic fibrosis isolates has been investigated by Bakkal et al. (2010). A study of *B. pseudomallei* antagonism displayed by *B. ubonensis* provided indications of the production of a pepsin-sensitive bacteriocin-like compound (Marshall et al. 2010). However, antagonistic molecules involved in these interactions have not been identified yet.

Bacteriocins have the potential of selectively killing target cells and some of these molecules deserve further scrutiny as candidate alternative antibacterials (Brown et al. 2012; Lukacik et al. 2012; Riley et al. 2012; Cotter et al. 2013). Here, we report on the bacteriocin activity of a lectin-like protein encoded in the genome of a *B. cenocepacia* human isolate.

## MATERIALS AND METHODS

### Strains and culture conditions

Bacterial strains and plasmids used in this study are listed in Table S1. *Bordetella*,* Escherichia coli,* and *Ralstonia* were routinely grown in shaken LB broth (MP Biomedicals, Brussels, Belgium) at 37°C. *Burkholderia* strains were grown in LB broth or Tryptic Soy Broth (TSB, BD Biosciences, Erembodegem, Belgium), at 37°C with shaking. *Achromobacter*,* Pseudomonas,* and *Xanthomonas* were grown in TSB, *Alcaligenes* in 869 medium, *Azoarcus* in medium 1 LMG, *Chromobacterium* and *Variovorax* in LB, and *Herbaspirillum* in Nutrient Broth, at 30°C with shaking. Alternative media to initiate LlpA production in *B. cenocepacia* AU1054 are listed in Table S2. Media were solidified with 1.5% agar (Invitrogen, Ghent, Belgium) and supplemented with filter-sterilized kanamycin (Sigma-Aldrich, Diegem, Belgium) at 50 μg/mL when required.

Plasmids used for sequencing were propagated in *E. coli* TOP10F' (Invitrogen). *E. coli* BL21 (DE3) (Novagen, Darmstadt, Germany) was taken as a host for recombinant protein expression. Genomic DNA from *Burkholderia* strains was isolated using the Puregene Yeast/Bact. Kit B (Qiagen, Venlo, Netherlands). Plasmid DNA was extracted using the QIAprep Spin Miniprep Kit (Qiagen). Bacterial stocks were stored at −80°C in the appropriate medium in 25% (v/v) glycerol.

### Recombinant DNA methods

Standard methods were used for the preparation of competent *E. coli* cells and heat shock transformation of *E. coli* (Green and Sambrook 2012). DNA ligation was performed using T4 DNA ligase (Invitrogen). Restriction enzymes were used according to the supplier's specifications (Roche Diagnostics, Vilvoorde, Belgium). Plasmid sequencing was performed by GATC Biotech (Constance, Germany).

*Burkholderia llpA* genes were amplified by polymerase chain reaction (PCR) with Platinum *Pfx* DNA polymerase (Invitrogen), using a C1000 Thermal Cycler (Bio-Rad, Nazareth Eke, Belgium). Genomic DNA from *B. cenocepacia* AU1054 and *B. ambifaria* MEX-5 was taken as a template; PCR primers are listed in Table S3. Amplicons were purified using the QIAquick PCR Purification Kit (Qiagen), digested with NdeI and XhoI*,* ligated in pET28a(+), and transformed to *E. coli* TOP10F'. Transformants were verified for the presence of insert by PCR using *Taq* Polymerase (BIOKÉ, Leiden, Netherlands) with primers PGPRB-5104 and PGPRB-5105. Insert confirmed plasmids (pCMPG6192 (Bcen_1091) and pCMPG6196 (Bcen_1092) from *B. cenocepacia* AU1054; pCMPG6200 (Bamb_0926) from *B. ambifaria* MEX-5) were purified and checked by sequencing. The three *llpA* genes were cloned without their predicted N-terminal secretory motifs (SignalP; http://www.cbs.dtu.dk/services/SignalP), with the His_6_-tag at the N-terminus (Parret et al. 2004; Ghequire et al. 2012a).

### Overexpression and purification of recombinant *B. cenocepacia* LlpA

Induction of expression, lysis of harvested cells by sonication, and protein extraction of N-terminal His-tagged LlpAs from *E. coli* BL21 (DE3), carrying expression constructs pCMPG6192, pCMPG6196, and pCMPG6200, have been described previously (Parret et al. 2004). Recombinant protein was purified by nickel affinity chromatography, using an Äkta Purifier (GE Healthcare Life Sciences, Amersham Bioscience, Diegem, Belgium) with a 5-mL HisTrap column (Ghequire et al. 2012a).

The presence of His-tagged protein was checked via immunodetection by Western blot, using monoclonal anti-His_6_ (IgG1 from mouse; Roche Diagnostics) as primary antibody. Fractions free of other proteins, as verified by SDS-PAGE and subsequent Coomassie Blue staining, were dialyzed against bis-TRIS propane buffer (20 mmol/L, 200 mmol/L NaCl, pH 7.0). Concentrations of purified recombinant proteins were determined by absorbance measurement at 280 nm with molar extinction coefficients of 56,505 mol/L^−1^ cm^−1^ for Bcen_1091 (with calculated molecular weight 30,566 Da), 58,120 mol/L^−1^ cm^−1^ for Bcen_1092 (29,144 Da), and 74,620 mol/L^−1^ cm^−1^ for Bamb_0926 (30,046 Da) (Pace et al. 1995). N-terminal amino acid sequences of proteins on a blot were determined by Edman degradation, using a Procise 491 cLC protein sequencer (Applied Biosystems, Foster City, CA).

### Bacteriocin assay

Antibacterial activity of purified recombinant His-tagged protein was detected by spot assay (Ghequire et al. 2012a). Briefly, plates were overlaid with 5 mL of soft agar (0.5%) of the appropriate medium, seeded with 50 μL of an indicator culture (16 h, ~10^8^–10^9^ CFU/mL) to generate a cell lawn. After drying, a 10-μL spot of purified recombinant protein (conc. 1 mg/mL) was applied on the plate. Bis-TRIS propane buffer was used as a negative control. Plates were incubated overnight at 30°C or 37°C (depending on the strains tested) and evaluated for the presence of zones of growth inhibition (halos) next day.

### Determination of the MIC of LlpA

The minimum inhibitory concentration (MIC) of LlpA was determined in triplicate following the broth microdilution procedure using flat-bottom 96-well microtiter plates (TPP, Trasadingen, Switzerland) (European committee for antimicrobial susceptibility testing (EUCAST) 2003; Brackman et al. 2009). Briefly, an overnight inoculum (TSB medium, 24 h) of *B. ambifaria* LMG 19182 was diluted to ~5.10^5^ CFU/mL, supplemented with a twofold dilution series of recombinant His_6_-tagged LlpA (Bcen_1091), and incubated overnight at 37°C (20 h). Bis-TRIS propane buffer was used as a control. The MIC was determined as the minimum concentration of LlpA at which no growth of the indicator strain was observed (OD_590_ <0.1) after 24 h of incubation.

### Determination of the inhibition and eradication of *Burkholderia* biofilms

The minimal biofilm inhibitory concentration (MBIC) of LlpA was determined by assaying the growth-inhibitory effect on freshly adhered sessile cells (Peeters et al. 2009). In brief, an overnight culture was diluted to ~10^8^ CFU/mL and the suspension was added to the wells of a round-bottomed 96-well microtiter plate (TPP). After 4 h of adhesion, the supernatant was removed and the plates were rinsed with physiological saline (PS, 0.9% NaCl). One hundred microliter volumes of LlpA at selected concentrations (in bis-TRIS propane buffer) were added to the wells and the plates were further incubated at 37°C. After 20 h of treatment, wells were rinsed a final time with PS. Cells were removed from the wells of the plates by vortexing and sonication, and suspended in 100-μL volumes of PS. The number of CFU/biofilm was determined by plating the resulting suspension on Tryptic Soy Agar (TSA). Results were averaged over 8 technical repeats. The MBIC was defined as the minimum concentration of LlpA necessary to prevent biofilm growth and maturation.

To determine the biofilm eradication effect of LlpA, biofilms of *B. ambifaria* were initially grown in the wells of round-bottomed 96-well plates (Brackman et al. 2009). After formation of the biofilm (24 h), planktonic cells were washed away by rinsing the plates with PS. Subsequently, 100-μL volumes of LlpA at selected concentrations (in bis-TRIS propane buffer) were added to interact with the biofilm, submerged in 100-μL volumes of PS. Plates were incubated for another 24 h and rinsed again with PS. Sessile cells were removed from the wells of the plates by vortexing and sonication, suspended in 100-μL volumes of PS. The number of CFU/biofilm was determined by plating the resulting suspension on TSA. Results were averaged over 16 repeats (2 biological repeats, 8 technical repeats) (Brackman et al. 2011).

### Phylogenetic analysis

Putative LlpA-like proteins in *Burkholderia* spp. were identified using the National Center for Biotechnology (NCBI) non-redundant database, via Blast searches. Sequences of previously identified LlpAs from *Pseudomonas* and *Xanthomonas* were used as a query. Multiple sequence alignments and phylogenetic analysis were performed with the Geneious 5.4 software (Drummond et al. 2011). Predicted N-terminal signal peptide sequences (SignalP), if present, were removed before alignment.

## Results

### Genes encoding LlpA-like proteins in *Burkholderia* genomes

Lectin-like bacteriocins, called LlpAs, represent a novel class of antibacterial proteins identified in the γ-proteobacterial genera *Pseudomonas* and *Xanthomonas* (Ghequire et al. 2012b). Being composed of a MMBL (monocot mannose-binding lectin) domain tandem, they show remarkable structural similarities with plant lectins (Ghequire et al. 2013). The prototypical MMBL domain is characterized by the presence of three potential mannose-binding pockets, identifiable by an amino acid stretch corresponding to the motif QxDxNxVxY (with x representing any amino acid). Carbohydrate-binding capacity of the C-terminal domain of LlpA is crucial for killing, whereas the N-terminal domain represents the main specificity determinant (Ghequire et al. 2013).

Through homology searches we identified several genes encoding potential new members of the LlpA family in genomic sequences of the genus *Burkholderia*. Amino acid sequence alignment of the derived gene products with functionally characterized LlpA bacteriocins from pseudomonads and xanthomonads, displayed the characteristic tandem MMBL organization, although the level of amino acid sequence identity with these LlpAs is low (<30%; Fig. S1). The second MMBL domain is followed by an equivalent of the β-hairpin in *Pseudomonas putida* LlpA (Ghequire et al. 2013), a poorly conserved carboxyterminal extension varying considerably in length (from 24 to 52 amino acids) among different *Burkholderia* strains. The presence of a typical aminoterminal signal sequence suggests that the *Burkholderia* proteins are proteolytically processed upon Sec-dependent secretion, similarly to LlpA4 translocation in *Xanthomonas* but differently from the unknown export route of pseudomonad LlpA proteins, which all lack a cleavable aminoterminal signal peptide (Ghequire et al. 2012b, 2013).

**Figure 1:**
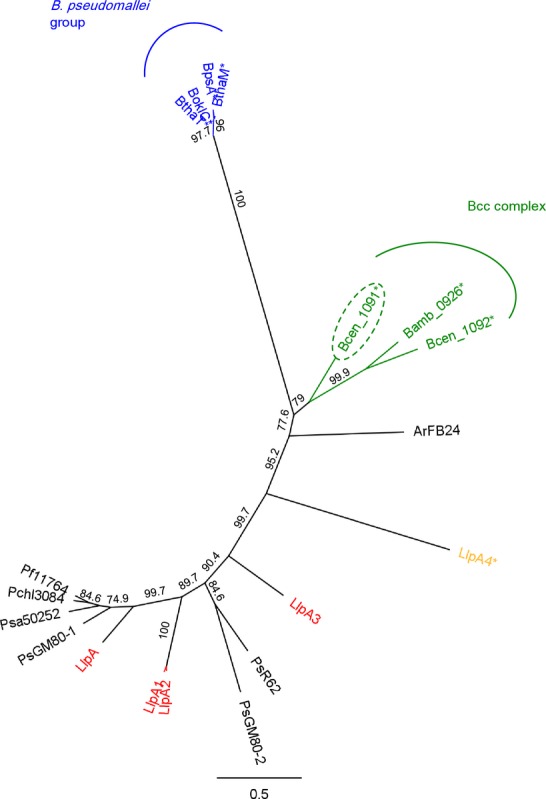
Phylogenetic analysis of LlpA-like proteins. Unrooted maximum-likelihood tree (PhyML; JTT matrix, [Guindon and Gascuel 2003]) inferred from a multiple amino acid alignment (Fig. S1) of bacteriocins from *Pseudomonas putida* BW11M1 (LlpA, [Parret et al. 2003]), *P. fluorescens* Pf-5 (LlpA1 = PFL_1229, LlpA2 = PFL_2127 [*P. fluorescens* Wayne1, *CADW01000205*], [Parret et al. 2005]), *P. syringae* pv. *syringae* 642 (LlpA3, [Ghequire et al. 2012a]), and *Xanthomonas axonopodis* pv. *citri* str. 306/*Xanthomonas citri* pv. *malvacearum *LMG 761 (LlpA4 [*X. axonopodis* pv. *malvacearum *GSPB1386, *AHIB01000114*], [Ghequire et al. 2012a]), with hypothetical proteins from *Arthrobacter* sp. FB24 (ArFB24, YP_829274), *Burkholderia ambifaria *MEX-5 (Bamb_0926, ZP_02905572), *B. cenocepacia* AU1054 (Bcen_1091, ABF75998; Bcen_1092, ABF75999 [strains MC0-3, HI2424]), *B. oklahomensis* C6786 (BoklC, ZP_02366769 [strain EO147], *B. pseudomallei* 1710a (BpsA, ZP_04953366 [46 strains]), *B. thailandensis *TXDOH (BthaT, ZP_02369690 [strains Bt4, E264]), and *B. thailandensis *MSMB43 (BthaM, ZP_02467384), *Pseudomonas chlororaphis* subsp. *aureofaciens* 30-84 (Pchl3084, ZP_18874430), *P. fluorescens *NCIMB 11764 (Pf11764, *ALWP01000740*), *P. syringae* pv. *aptata *DSM 50252 (Psa50252, EGH77666), *Pseudomonas* sp. GM80 (PsGM80-1, ZP_10606046; PsGM80-2, ZP_10606131), and *Pseudomonas* sp. R62 (PsR62, *AHZM01000533*). Orthologues (99–100% amino acid identity) of the listed proteins are mentioned in square brackets (sequences not included in Fig. S1 and tree). Before alignment, predicted N-terminal signal peptide sequences (SignalP), if present, were removed (names marked with asterisk). Underlined accession numbers represent unannotated nucleotide sequences from unfinished microbial genomes encoding LlpA-like proteins identified by Blast searches. The scale bar represents 0.5 substitutions per site. Bootstrap values (percentage of 1000 replicates) are shown at the branches. Values lower than 50 are not displayed. LlpA from *B. cenocepacia* AU1054, subject of this study, is indicated by a dotted ellipse. Previously characterized LlpAs from γ-proteobacteria are indicated in red (*Pseudomonas*) and orange (*Xanthomonas*). Nodes of LlpA-like proteins from *Burkholderia* belonging to the Bcc complex are colored in green and those from the *B. pseudomallei* group in blue.

**Figure 2:**
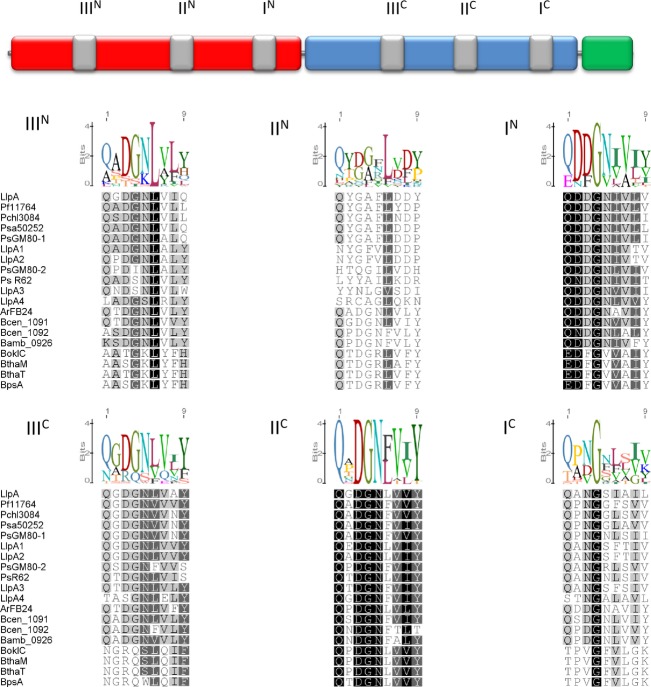
General domain structure of LlpA proteins with potential carbohydrate-binding motifs (gray). The N-domain is colored red, the C-domain blue, and the C-terminal extension green. The respective potential mannose-binding motifs corresponding to the consensus motif QxDxNxVxY in LlpA-like proteins (derived from sequence alignment in Fig. S1) are aligned. The protein codes and accession numbers are specified in Figure 1. Sequence conservation is visualized by differential shading and the sequence logo graph visualizes the degree of consensus for each residue.

Phylogenetic comparison of the LlpA-like *Burkholderia* proteins revealed two distinct clusters, with separate grouping of sequences from four Bcc strains (*B. ambifaria* MEX-5, and *B. cenocepacia* AU1054, HI2424, and MC0-3) and those from a large number of *B. pseudomallei* group strains: *Burkholderia oklahomensis* C6786 (with an orthologue in strain EO147), *B. pseudomallei* (with orthologues in >45 strains), *B. thailandensis* MSMB4, and *B. thailandensis* TXDOH (with orthologues in strains Bt4 and E264) (Fig. 1). As the *B. pseudomallei* group sequences share high amino acid sequence identity (>87%), essentially four different types of LlpA-like proteins can be distinguished. The three Bcc types share 43–60% mutual identity but show poor homology with the conserved *B. pseudomallei* group sequences, apart from the presence of MMBL signature motifs (Fig. S1; Fig. 2). In contrast to the *B. pseudomallei* group coding sequences (~64% G+C), the GC content of the Bcc genes (50–55% G+C) is considerably lower than the respective genomic values (66–67% G+C). An additional indication for acquisition by lateral gene transfer is the nearby presence of a tRNA gene and integrase in the *B. cenocepacia* genomes.

The phylogenetic tree further shows that the Bcc LlpA-like protein sequences are more similar to the bacteriocins from γ-proteobacteria than to the proteins found in the *B. pseudomallei* group. This is also apparent from the relative sequence conservation of the QxDxNxVxY motif corresponding to the six potential carbohydrate-binding pockets, three in each MMBL domain (Fig. 2). Best conserved across all known sequences, including those of the *B. pseudomallei* group, is pocket II^C^ for which an additive role in bacteriocin toxicity of *P. putida* LlpA was demonstrated (Ghequire et al. 2013). However, unlike the Bcc proteins, the *B. pseudomallei* group sequences lack motif conservation equivalent to the crucial carbohydrate-binding site III^C^ in *P. putida* LlpA. A similar dichotomy among the *Burkholderia* sequences is evident for three LlpA sites lacking carbohydrate-binding capacity (III^N^, I^N^, I^C^). Whereas the canonical mannose-binding motif II^N^ is essentially absent in pseudomonad LlpAs, this stretch is well conserved in the burkholderiad sequences. These observations suggest that carbohydrate-binding properties of the *Burkholderia* LlpA-like proteins may differ between both groups, but also diverge from the *P. putida* prototype LlpA.

### Bacteriocin activity of *B. cenocepacia* LlpA

**Figure 3:**
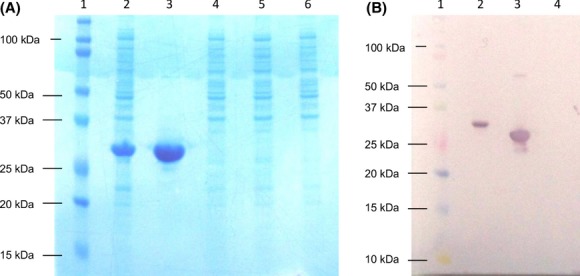
Purification and activity of recombinant *Burkholderia cenocepacia* AU1054 LlpA. (A) SDS-PAGE analysis of His-tagged Bcen_1091 expression and purification. Proteins present in the lysate soluble fraction of *Escherichia coli* BL21 (DE3) cells carrying the specified plasmids. Lane 1: Kaleidoscope protein ladder; lane 2, lysate of pCMPG6192 (Bcen_1091 from *B. cenocepacia* AU1054); lane 3, purified LlpA (Bcen_1091 from *B. cenocepacia* AU1054); lane 4, lysate of pCMPG6196 (Bcen_1092 from *B. cenocepacia* AU1054); lane 5, lysate of pCMPG6200 (Bamb_0926 from *B. ambifaria* MEX-5); and lane 6, lysate of pET28a(+) (no insert). (B) Western Blot analysis with anti-LlpA antibodies. Lane 1: Kaleidoscope protein ladder; lane 2: 2.5 ng of purified *Pseudomonas* LlpA (Parret et al. 2004); lane 3: 2.5 ng of purified *Burkholderia* LlpA; lane 4: 2.5 ng of bovine serum albumin (negative control).

To enable testing of the potential antibacterial activity of the Bcc LlpA-like proteins, heterologous expression in *E. coli* was attempted. The *B. cenocepacia* AU1054 genes encoding Bcen_1091 and Bcen_1092, and Bamb_0926 from *B. ambifaria* MEX-5 were cloned into pET28a(+) with an N-terminal His_6_-tag immediately fused to the mature protein, lacking the respective predicted signal peptides for Sec-dependent secretion (Fig. S1). Detectable expression could only be achieved for Bcen_1091 (Fig. 3A). The protein was purified by Ni-NTA affinity chromatography (Fig. 3A) and its identity was confirmed by N-terminal sequencing and Western Blotting with anti-His_6_ antibodies. The *Burkholderia* LlpA was found to be cross-reactive with antibodies raised against LlpA_BW11M1_ from *P. putida* (Fig. 3B; Parret et al. 2005). The purified LlpA showed no detectable binding to glycan array slides (Fig. S2, Blixt et al. 2004). This was also observed for *Pseudomonas* LlpAs (Ghequire et al. 2012a, 2013; M. Ghequire and R. De Mot, unpubl. data) and may be due to the absence of appropriate prokaryotic target carbohydrates.

Bacteriocin activity was assayed by applying spots of pure protein on indicator-seeded agar plates (Ghequire et al. 2012a) to visualize growth inhibition as a halo formed in a confluent lawn of bacterial target cells. The test panel included representative members of the Bcc complex and clinical isolates belonging to the *B. pseudomallei* group (Table 1). In addition, a set of other β-proteobacteria and several γ-proteobacteria (*Pseudomonas* and *Xanthomonas* spp.) were tested (Table S1). Antibacterial activity was only observed against strains belonging to the Bcc complex (16.7% of strains in the test panel). Whereas none of the *B. pseudomallei* group strains and only one of the *B. cenocepacia* strains proved susceptible, several *B. ambifaria* strains were found to be sensitive to the LlpA-like protein. Such patchy strain-dependent pattern of sensitivity is in line with the activity spectra of the corresponding *Pseudomonas* and *Xanthomonas* bacteriocins (Parret et al. 2003, 2005; Ghequire et al. 2012a). Other β-proteobacteria were not susceptible, nor were the tested γ-proteobacteria affected, confirming that this Bcc LlpA is a narrow-spectrum genus-specific bacteriocin that acts across species borders.

**Table:** Antibacterial activity of purified recombinant LlpA (Bcen_1091 from *Burkholderia cenocepacia* AU1054) against a panel of *Burkholderia* strains

Indicator strain		Growth inhibition by LlpA	Indicator strain		Growth inhibition by LlpA
**Bcc complex**			*B. seminalis*	LMG 24272	−
*B. ambifaria*	LMG 17828	+	*B. stabilis*	LMG 14294	−
	LMG 17829	+		LMG 14086	−
	LMG 19182	+	*B. ubonensis*	LMG 20358	−
	LMG 19466	+		LMG 24263	−
	LMG 19467	−	*B. vietnamensis*	LMG 18835	−
	LMG 26702	−		LMG 10927	−
*B. anthina*	LMG 20980	+		LMG 10929	−
	LMG 20983	−			
*B. arboris*	LMG 24066	−	***B. Pseudomallei*** **group**		
	R-132	−	*B. mallei*	NCTC 120	−
*B. cenocepacia*	LMG 6986	−	*B. mallei*	NCTC 3708	−
	LMG 16656	−	*B. mallei*	NCTC 3709	−
	LMG 16659	−	*B. mallei*	NCTC 10229	−
	LMG 18826	−	*B. mallei*	NCTC 10230	−
	LMG 18827	−	*B. mallei*	NCTC 10245	−
	LMG 18828	−	*B. mallei*	NCTC 10247	−
	LMG 18829	−	*B. mallei*	NCTC 10248	−
	LMG 18830	−	*B. mallei*	NCTC 10260	−
	LMG 18863	−	*B. pseudomallei*	ATCC11668	−
	LMG 19230	+	*B. pseudomallei*	Bengla 01	−
	LMG 21461	−	*B. pseudomallei*	ID 1476	−
	LMG 21462	−	*B. pseudomallei*	NCTC 1688	−
*B. cepacia*	LMG 1222	−	*B. pseudomallei*	NCTC 4845	−
	LMG 18821	−	*B. pseudomallei*	NCTC 4846	−
*B. contaminans*	LMG 16227	−	*B. pseudomallei*	NCTC 6700	−
	R-12710	+	*B. pseudomallei*	NCTC 7383	−
*B. diffusa*	LMG 24065	−	*B. pseudomallei*	NCTC 7431	−
	LMG 24266	−	*B. pseudomallei*	NCTC 8016	−
*B. dolosa*	LMG 18941	−	*B. pseudomallei*	NCTC 8707	−
	LMG 18943	−	*B. pseudomallei*	NCTC 8708	−
*B. lata*	LMG 6992	−	*B. pseudomallei*	NCTC 10274	−
	R-9940	−	*B. pseudomallei*	NCTC 10276	−
*B. latens*	LMG 24064	−	*B. pseudomallei*	NCTC 11642	−
	R-11768	−	*B. pseudomallei*	UCl 467	−
*B. metallica*	LMG 24068	+	*B. thailandensis*	CIP 106301	−
	R-2712	−	*B. thailandensis*	CIP 106302	−
*B. multivorans*	LMG 18825	−			
	LMG 13010	−	**Other strains**		
*B. pyrrocinia*	LMG 14191	−	*B. glumae*	LMG 2196	−
	LMG 21824	−	*B. gladioli*	LMG 2216	−
*B. seminalis*	LMG 24067	−	*B. plantarii*	LMG 9035	−

### Antibiofilm activity of *B. cenocepacia* LlpA

The MIC for planktonic cells was determined using a microdilution assay. For *B. ambifaria* LMG 19182, a representative strain from the indicator panel, a MIC value of 7.0 μg/mL (0.23 μmol/L) was obtained. Previously identified compounds-mediating antagonism between *Burkholderia* species, enacyloxin IIa and capistruin, displayed MIC values of 9.3 and 12 μmol/L, respectively (Knappe et al. 2008; Mahenthiralingam et al. 2011), 40- to 50-fold higher than LlpA.

Possible antibiofilm activity of LlpA against strain LMG 19182 was studied by monitoring the biofilm inhibitory and eradicatory effects at super- and sub-MIC concentrations. Treatment at 5.8 μmol/L LlpA (25 × MIC) at the onset of biofilm formation (biofilm inhibition) resulted in a 52% reduction of the biofilm, whereas at 58 nmol/L LlpA (0.25 × MIC) a 36% reduction could be observed. Addition of LlpA to a mature biofilm (biofilm eradication) reduced CFU count by 62% (with a standard deviation of 24%) when LlpA was applied at a concentration of 5.8 μmol/L. No CFU reduction was observed at 58 nmol/L LlpA.

## Discussion

Whereas numerous bacteriocins from other Proteobacteria, such as *E. coli* and *Pseudomonas*, have been characterized, this article constitutes the first description of such a protein-mediating antagonism among members of the *Burkholderia* genus. This lectin-like protein from *B. cenocepacia* AU1054 displays an intrinsic killing activity significantly higher than the previously identified *Burkholderia* molecules with intragenus toxicity, the polyketide enacyloxin IIa, and the lasso peptide capistruin. Notably, the *B. cenocepacia* protein was also active against sessile cells growing in a biofilm. Gram-negative bacteriocins exerting killing activity on biofilm cells have also been described for *Pseudomonas aeruginosa* (Smith et al. 2012) and *Citrobacter freundii* (Shanks et al. 2012). As observed for several other bacteriocins from Gram-negative bacteria, the susceptibility to this *B. cenocepacia* protein is highly strain dependent, making it not readily suitable as an alternative for antibiotics. As strain selectivity and killing activity appear to be linked with different parts of the LlpA protein (Ghequire et al. 2013), insight in its mode of action may nevertheless reveal alternative targets for killing bacteria. As the *Burkholderia* LlpA-like proteins differ from the pseudomonad LlpAs in relative sequence conservation of the potential carbohydrate-binding pockets present in both MMBL domains, determinants of toxicity and selectivity may also deviate from those identified in the prototype *P. putida* LlpA (Ghequire et al. 2013).

This type of antibacterial protein likely plays a role in the social life of *Burkholderia* strains in their natural environment by mediating antagonism among strains colonizing similar niches. Expression of the tandemly organized *llpA*-like genes from human isolate *B. cenocepacia* AU1054 and soil isolate *B. cenocepacia* HI2424, both belonging to the PHDC lineage (LiPuma et al. 2002), was observed by RNA-seq, both in conditions mimicking a soil environment and in synthetic cystic fibrosis sputum medium (Yoder-Himes et al. 2009). However, our attempts to trigger detectable bacteriocin production on different media, including rhizosphere soil extract media (Table S2), were not successful (data not shown).

Among bacteriocins, LlpA takes a unique position by virtue of its lectin-like properties. A number of “genuine” lectins with different carbohydrate-binding specificities have been identified in *B. cenocepacia* (Lameignere et al. 2010; Sulák et al. 2011) and *B. ambifaria* (Audfray et al. 2012). A possible role in biofilm formation has been proposed for them (Inhülsen et al. 2012), but they have no known antimicrobial activities. Conceivably, the more distantly related MMBL-like proteins from the *B. pseudomallei* group representatives may have evolved toward a similar biofilm-supporting function by promoting physical interbacterial interaction rather than antagonism. Compared to the Bcc cluster proteins which harbor bacteriocin activity, those of the *B. pseudomallei* group show little sequence divergence, have conserved a different set of potential carbohydrate pockets, and are encoded by genes lacking characteristics of (recent) acquisition, suggesting a more general, possibly virulence-related physiological role, although the latter remains speculative at this point.

## Supporting Information

**Table S1.** List of strains and plasmids used in this study. **Table S2.** List of bacterial growth media tested to trigger LlpA production in *B. cenocepacia* AU1054. Media were solidified with 1.5% agar. Overlay assays with LlpA-sensitive bacteria after chloroform killing of *B. cenocepacia* AU1054 were done in LB soft agar (0.5% agar). Media were also tested in coculture, in the latter case cell lawns of indicator bacteria were made in soft agar, after which AU1054 was spotted on top. In parallel, the different growth media were also tested when *B. cenocepacia* AU1054 was supplemented with mitomycin (1 μg/mL final concentration) prior to spotting. Plates were scored after overnight incubation at 37°C. Generated halos were not pronase sensitive, and not thought to be bacteriocin derived. **Table S3.** List of primers used in this study. **Figure S1.** Multiple amino acid sequence alignment used to construct the phylogenetic tree of LlpA homologues (Fig. ). The protein codes and accession numbers are specified in Figure 1. Sequence conservation is visualized by differential shading. The position of the sequences corresponding to QxDxNxVxY-like motifs of MMBL proteins, representing potential carbohydrate-binding pockets, and the separate domains (N- and C-domain with MMBL fold; β-hairpin extension) present in *P. putida* BW11M1 LlpA (PDB 3M7H) are indicated. **Figure S2.** Glycan array profile of LlpA (Bcen_1091 from *B. cenocepacia* AU1054) as measured by fluorescence intensity. A complete list of tested carbohydrates (array version PA v5) is available from the Consortium of Functional Glycomics (CFG, www.functionalglycomics.org).
