# Supplementary material for: Antibacterial activity of a lectin-like Burkholderia cenocepacia protein
Source: Microbiologyopen. 2013 Jun 5;2(4):566–75. doi: 10.1002/mbo3.95 (PMC3831624; doi:10.1002/mbo3.95)
Supplement: Supplementary file 1 — Table S1. List of strains and plasmids used in this study. Table S2. List of bacterial growth media tested to trigger LlpA production in B. cenocepacia AU1054. Media were solidified with 1.5% agar. Overlay assays with LlpA-sensitive bacteria after chloroform killing of B. cenocepacia AU1054 were done in LB soft agar (0.5% agar). Media were also tested in coculture, in the latter case cell lawns of indicator bacteria were made in soft agar, after which AU1054 was spotted on top. In parallel, the different growth media were also tested when B. cenocepacia AU1054 was supplemented with mitomycin (1 μg/mL final concentration) prior to spotting. Plates were scored after overnight incubation at 37°C. Generated halos were not pronase sensitive, and not thought to be bacteriocin derived. Table S3. List of primers used in this study. Figure S1. Multiple amino acid sequence alignment used to construct the phylogenetic tree of LlpA homologues (Fig. ). The protein codes and accession numbers are specified in Figure 1. Sequence conservation is visualized by differential shading. The position of the sequences corresponding to QxDxNxVxY-like motifs of MMBL proteins, representing potential carbohydrate-binding pockets, and the separate domains (N- and C-domain with MMBL fold; β-hairpin extension) present in P. putida BW11M1 LlpA (PDB 3M7H) are indicated. Figure S2. Glycan array profile of LlpA (Bcen_1091 from B. cenocepacia AU1054) as measured by fluorescence intensity. A complete list of tested carbohydrates (array version PA v5) is available from the Consortium of Functional Glycomics (CFG, www.functionalglycomics.org). [file mbo30002-0566-SD1.docx]

**Supporting information**

**Antibacterial activity of a lectin-like**

***Burkholderia cenocepacia* protein**

Maarten G. K. Ghequire^1^, Evelien De Canck^2^, Pierre Wattiau^3^, Iris Van Winge^1^,

Remy Loris^4,5^, Tom Coenye^2^, René De Mot^1^*

^1^ Centre of Microbial and Plant Genetics, University of Leuven, Kasteelpark Arenberg 20 box 2460, 3001 Heverlee, Belgium

^2^ Laboratory of Pharmaceutical Microbiology, Ghent University, 9000 Ghent, Belgium

^3^ Department of Bacteriology and Immunology, Veterinary and Agrochemical Research Centre, 1180 Brussels, Belgium

^4^ Molecular Recognition Unit, Department of Structural Biology, Vlaams Instituut voor Biotechnologie, 1050 Brussels, Belgium

^5^ Structural Biology Brussels, Department of Biotechnology (DBIT), Vrije Universiteit Brussel, 1050 Brussels, Belgium

* For correspondence. E-mail: [rene.demot@biw.kuleuven.be](mailto:rene.demot@biw.kuleuven.be); Tel. +32 (0)16 329681, Fax: +32 (0)16 321963

**Table S1.** List of strains and plasmids used in this study.

|  | **Bacteria** | **Strain** | **Genotype or relevant characteristic(s)** | **Source or reference** |
| --- | --- | --- | --- | --- |
| **β-proteobacteria** | |  |  |  |
| **Bcc *Burkholderia*** | |  |  |  |
|  | *B. ambifaria* | LMG 17828 | Type strain, corn roots isolate, USA | BCCM^a^ |
|  |  | LMG 17829 | Corn roots isolate, USA | BCCM |
|  |  | LMG 19182 | Type strain, isolated from pea rhizosphere, USA | BCCM |
|  |  | LMG 19466 | Isolate from cystic fibrosis (CF) patient, USA | BCCM |
|  |  | LMG 19467 | Isolate from CF patient, Australia | BCCM |
|  |  | LMG 26702 |  | BCCM |
|  | *B. anthina* | LMG 20980 | Type strain, soil rhizosphere isolate, USA | BCCM |
|  |  | LMG 20983 | Sputum isolate from CF patient, UK | BCCM |
|  | *B. arboris* | LMG 24066 | Type strain, soil isolate, USA | BCCM |
|  |  | R-132 | Isolate from CF patient, USA | P. Vandamme^b^ |
|  | *B. cenocepacia* | LMG 6986 | Urine isolate, USA | BCCM |
|  |  | LMG 16656 | Type strain, sputum isolate from CF patient, UK | BCCM |
|  |  | LMG 16659 | Isolate from CF patient, UK | BCCM |
|  |  | LMG 18826 | Isolate from CF patient, Canada | BCCM |
|  |  | LMG 18827 | Isolate from CF patient, Canada | BCCM |
|  |  | LMG 18828 | Isolate from CF patient, Canada | BCCM |
|  |  | LMG 18829 | Isolate from CF patient, USA | BCCM |
|  |  | LMG 18830 | Isolate from CF patient, Australia | BCCM |
|  |  | LMG 18863 | Isolate from CF patient, Canada | BCCM |
|  |  | LMG 19230 | Isolate from wheat root endophyte, Australia | BCCM |
|  |  | LMG 21461 | Sputum isolate from CF patient, Argentina | BCCM |
|  |  | LMG 21462 | Sputum isolate from CF patient, Italy | BCCM |
|  | *B. cepacia* | LMG 1222 | Type strain, isolate from *Allium cepa*, USA | BCCM |
|  |  | LMG 18821 | Isolate from CF patient, Australia | BCCM |
|  | *B. contaminans* | LMG 16227 | Isolate from respiratory tract from CF patient, Sweden | BCCM |
|  |  | R-12710 | Type strain, milk isolate from sheep with mastitis, Spain | BCCM |
|  | *B. diffusa* | LMG 24065 | Type strain, isolate from CF patient, USA | BCCM |
|  |  | LMG 24266 | Isolate from CF patient, Canada | BCCM |
|  | *B. dolosa* | LMG 18941 | Isolate from CF patient, USA | BCCM |
|  |  | LMG 18943 | Isolate from CF patient, USA | BCCM |
|  | *B. lata* | LMG 6992 | Soil isolate, Trinidad and Tobago | BCCM |
|  |  | R-9940 | Isolate from CF patient, Canada | P. Vandamme |
|  | *B. latens* | LMG 24064 | Type strain, isolate from CF patient, Italy | BCCM |
|  |  | R-11768 | Isolate from CF patient, UK | P. Vandamme |
|  | *B. metallica* | LMG 24068 | Type strain, isolate from CF patient, USA | BCCM |
|  |  | R-2712 | Isolate from CF patient, Canada | P. Vandamme |
|  | *B. multivorans* | LMG 18825 | Isolate from CF patient, UK | BCCM |
|  |  | LMG 13010 | Isolate from CF patient, UK | BCCM |
|  | *B. pyrrocinia* | LMG 14191 | Type strain, soil isolate | BCCM |
|  |  | LMG 21824 | Isolate from CF patient, USA | BCCM |
|  | *B. seminalis* | LMG 24067 | Type strain, isolate from CF patient, USA | BCCM |
|  | *B. seminalis* | LMG 24272 | Isolate from nosocomial infection, Thailand | BCCM |
|  | *B. stabilis* | LMG 14294 | Type strain, isolate from CF patient, Belgium | BCCM |
|  |  | LMG 14086 | Isolated from a respirator, UK | BCCM |
|  | *B. ubonensis* | LMG 20358 | Type strain, isolated from surface soil, Thailand | BCCM |
|  |  | LMG 24263 | Isolate from nosocomial infection, Thailand | BCCM |
|  | *B. vietnamensis* | LMG 18835 | Isolate from CF patient, USA | BCCM |
|  |  | LMG 10927 | Isolate from *Oryza sativa* rhizosphere soil, Thailand | BCCM |
|  |  | LMG 10929 | Type strain, isolate from *Oryza sativa* rhizosphere soil, Vietnam | BCCM |
| ***Burkholderia pseudomallei* group** | |  |  |  |
|  | *B. mallei* | NCTC 120 |  | HPA^c^ |
|  | *B. mallei* | NCTC 3708 | Isolate from lung of mule, India | HPA |
|  | *B. mallei* | NCTC 3709 | Isolate from horse, India | HPA |
|  | *B. mallei* | NCTC 10229 | Isolate from bird | HPA |
|  | *B. mallei* | NCTC 10230 |  | HPA |
|  | *B. mallei* | NCTC 10245 | Isolate from lung and nose of horse | HPA |
|  | *B. mallei* | NCTC 10247 |  | HPA |
|  | *B. mallei* | NCTC 10248 | Isolate from human | HPA |
|  | *B. mallei* | NCTC 10260 | Isolate from human, Turkey | HPA |
|  | *B. pseudomallei* | ATCC11668 | Clinical isolate | ATCC^d^ |
|  | *B. pseudomallei* | Bengla 01 | Isolate from sick traveler from South Asia, Belgium | P. Wattiau^e^ |
|  | *B. pseudomallei* | ID 1476 | Isolate from sick traveler from South Asia, Belgium | P. Wattiau |
|  | *B. pseudomallei* | NCTC 1688 | Isolate from rat with melioidosis | HPA |
|  | *B. pseudomallei* | NCTC 4845 | Isolate from laboratory monkey | HPA |
|  | *B. pseudomallei* | NCTC 4846 | Isolate from laboratory monkey | HPA |
|  | *B. pseudomallei* | NCTC 6700 | Isolate from human | HPA |
|  | *B. pseudomallei* | NCTC 7383 |  | HPA |
|  | *B. pseudomallei* | NCTC 7431 |  | HPA |
|  | *B. pseudomallei* | NCTC 8016 | Isolate from sheep | HPA |
|  | *B. pseudomallei* | NCTC 8707 |  | HPA |
|  | *B. pseudomallei* | NCTC 8708 |  | HPA |
|  | *B. pseudomallei* | NCTC 10274 | Isolate from human blood, Malaysia | HPA |
|  | *B. pseudomallei* | NCTC 10276 | Isolate from human | HPA |
|  | *B. pseudomallei* | NCTC 11642 |  | HPA |
|  | *B. pseudomallei* | UCl 467 | Isolate from sick traveler from South Asia, Belgium | P. Wattiau |
|  | *B. thailandensis* | CIP106301 | Isolate from rice field soil, Thailand | IPC^f^ |
|  | *B. thailandensis* | CIP106302 |  | IPC |
| **Other *Burkholderia*** | |  |  |  |
|  | *B. glumae* | LMG 2196 | Type strain, isolate from *Oryza sativa* grain, Japan | BCCM |
|  | *B. gladioli* | LMG 2216 | Isolate from *Gladiolus* sp., USA | BCCM |
|  | *B. plantarii* | LMG 9035 | Type strain, isolate from *Oryza sativa* with blight and chlorosis, Japan | BCCM |
| **Non-burkholderiads** | |  |  |  |
|  | *Achromobacter xylosoxidans* | RW8P1 | Strain isolated from rice rhizosphere, Sri Lanka | Vlassak et al. 1992 |
|  | *Alcaligenes denitrificans* | PGSB 7792 | Isolate from maize rhizosphere, France | CMPG-collection |
|  | *Alcaligenes eutrophus* | AE81 |  | M. Mergeay^g^ |
|  | *Alcaligenes eutrophus* | AE215 |  | M. Mergeay |
|  | *Alcaligenes faecalis* | LMG 1229 | Type strain | BCCM |
|  | *Azoarcus indigens* | LMG 9092 | Isolate from Kallar grass, Pakistan | BCCM |
|  | *Bordetella avium* | 197N | Domesticated turkey isolate | Sebaihia et al. 2006 |
|  | *Chromobacterium violaceum* | CV026 | Mini-Tn*5* mutant of ATCC31532 | Throup et al. 1995 |
|  | *Herbaspirillum seropedicae* | LMG6513 | Type strain, isolate from *Oryza sativa* roots | BCCM |
|  | *Herbaspirillum seropedicae* | LMG6514 | Isolate from *Sorghum bicolor* roots | BCCM |
|  | *Ralstonia pickettii* | LMG 5942 | Type strain, isolate from tracheotomized patient, USA | BCCM |
|  | *Variovorax paradoxus* | LMG 1797 | Type strain, isolate from soil in mineral medium | BCCM |
|  |  |  |  |  |
| **γ-proteobacteria** | |  |  |  |
| ***Pseudomonas* group** | |  |  |  |
|  | *P. agarici* | LMG 2112 | Type strain, isolate from cultivated mushroom, New Zealand | BCCM |
|  | *P. chlororaphis* | LMG 5004 | Type strain, contaminated plate | BCCM |
|  | *P. fluorescens* | F113 | Isolate from sugarbeet rhizosphere | Shanahan et al. 1992 |
|  | *P. fluorescens* | 2-79 | Isolate from wheat rhizosphere | Weller and Cook 1983 |
|  | *P. fluorescens* | SBW25 | Isolate from sugarbeet leaves | Rainey and Bailey 1996 |
|  | *P. marginalis* pv. *marginalis* | LMG 2210 | Isolate from *Cichorium intybus*, Belgium | BCCM |
|  | *P. mendocina* | LMG 1223 | Type strain, isolate from ethanol enriched soil, Argentina | BCCM |
|  | *P. putida* | KT2440 | Restriction-deficient derivative of *Pseudomonas putida* mt-2 | Franklin et al. 1981 |
|  | *P. resinovorans* | LMG 2274 | Type strain, soil isolate, France | BCCM |
|  | *P. savastanoi* pv. *savastanoi* | LMG 5011 | Isolate from *Olea europaea*, Portugal | BCCM |
|  | *P. savastanoi* pv. *savastanoi* | LMG 2209 | Isolate from *Olea europaea*, Yugoslavia | BCCM |
|  | *P. syringae* pv. *glycinea* | LMG 5066 | Isolate from *Glycine max* with leaf blight, New Zealand | BCCM |
|  | *P. syringae* pv. *syringae* | LMG 1247 | Isolate from *Syringa vulgaris*, UK | BCCM |
|  | *P. tolaasii* | LMG 2342 | Type strain, isolate from cultivated mushroom, UK | BCCM |
|  | *P. tolaasii* | CH36 | Mushroom isolate | De Mot et al. 1994 |
|  | *P. viridiflava* | LMG 2352 | Type strain, isolate from *Phaseolus* sp., Switzerland | BCCM |
| ***Xanthomonas* group** | |  |  |  |
|  | *X. alfalfae* subsp. *alfalfae* | LMG 497 | Isolate from *Medicago sativa*, Sudan | BCCM |
|  | *X. axonopodis* pv. *manihotis* | LMG 629 | Leaf isolate from *Manihot esculenta*, Cameroon | BCCM |
|  | *X. axonopodis* pv. *manihotis* | LMG 784 | Isolate from *Manihot esculenta*, Brazil | BCCM |
|  | *X. campestris* pv*. campestris* | LMG 582 | Leaf isolate from *Brassica* sp., Belgium | BCCM |
|  | *X. citri* pv*. malvacearum* | LMG 761 | Isolate from *Gossypium* sp., Sudan | BCCM |
|  | *X. hortorum* pv*. hederae* | LMG 7411 | Isolate from *Hedera helix*, USA | BCCM |
|  | *X. translucens* | LMG 12921 | Callus isolate from *Anthurium andreanum* | BCCM |
|  | *X. translucens* pv*. graminis* | LMG 726 | Isolate from *Dactylis glomerata*, Switzerland | BCCM |
|  | *X. vasicola* pv*. holcicola* | LMG 736 | Isolate from *Sorghum bicolor*, New Zealand | BCCM |
|  | *X. vasicola* pv. *musacearum* | LMG 785 | Isolate from *Ensete ventricosum*, Ethiopia | BCCM |
|  | *X. vasicola* pv. *musacearum* | LMG 7431 | Isolate from *Musa* sp., Ethiopia | BCCM |
|  | *Xanthomonas* sp. pv. *zinniae* | LMG 8692 | Isolate from *Zinnia elegans*, Australia | BCCM |
|  |  |  |  |  |
| ***E. coli* strains** | |  |  |  |
|  | TOP10F’ |  | F'[*lac*I^q^ Tn*10*(Tet^R^)] *mcr*A Δ(*mrr-hsd*RMS*-mcr*BC) φ80*lac*ZΔM15 Δ*lac*X74 *rec*A1 *ara*D139 Δ(*ara-leu*)7697 *gal*U *gal*K *rps*L (Str^R^) *end*A1 *nup*G | Invitrogen |
|  | BL21(DE3) |  | F^–^ *ompT hsdS_B_*(r_B_^-^ m_B_^-^) *dcm* *gal* (DE3) | Novagen |
|  |  |  |  |  |
| **Plasmids** | |  |  |  |
|  | pET28a(+) |  | pBR322 origin, His-tag/thrombin/T7 tag ; Km^R^ | Novagen |
|  | pCMPG6192 |  | pET28a(+) with 858-bp PCR-amplified fragment containing Bcen_1091 from *B. cenocepacia* AU1054 cloned in NdeI/XhoI | This study |
|  | pCMPG6196 |  | pET28a(+) with 774-bp PCR-amplified fragment containing Bcen_1092 from *B. cenocepacia* AU1054 cloned in NdeI/XhoI | This study |
|  | pCMPG6200 |  | pET28a(+) with 786-bp PCR-amplified fragment containing Bamb_0926 from *B. ambifaria* MEX-5 cloned in NdeI/XhoI | This study |

^a^ BCCM: Belgian Coordinated Collections of Microorganisms (http://www.belspo.be/bccm/index.htm)

^b^ Peter Vandamme: Laboratory of Microbiology, University of Ghent, Ghent, Belgium

^c^ HPA: Health Protection Agency (http://www.hpa.org.uk/)

^d^ ATCC: American Type Culture Collection (http://www.lgcstandards-atcc.org/)

^e^ Pierre Wattiau: Department of Bacteriology and Immunology, Veterinary and Agrochemical Research Centre, Brussels, Belgium

^f^ IPC: Institut Pasteur Collection (http://www.crbip.pasteur.fr/)

^g^ Max Mergeay: Unit of Microbiology (MIC), Belgian Nuclear Research Centre, SCK.CEN, Mol, Belgium

**Table S2.** List of bacterial growth media tested to trigger LlpA production in *B. cenocepacia* AU1054. Media were solidified with 1.5 % agar. Overlay assays with LlpA-sensitive bacteria after chloroform killing of *B. cenocepacia* AU1054, were done in LB soft agar (0.5 % agar). Media were also tested in coculture, in the latter case cell lawns of indicator bacteria were made in soft agar, after which AU1054 was spotted on top. In parallel, the different growth media were also tested when *B. cenocepacia* AU1054 was supplemented with mitomycin (1 µg/ml final concentration) prior to spotting. Plates were scored after overnight incubation at 37°C. Generated halos were not pronase sensitive, not thought to be bacteriocin-derived.

| **Growth medium** | **Composition** |
| --- | --- |
| LB | 1 % bacterial peptone, 0.5 % yeast extract, 1 % NaCl |
| LB pH 5 | 1 % bacterial peptone, 0.5 % yeast extract, 1 % NaCl, adjustment to pH 5 |
| LB pH 9 | 1 % bacterial peptone, 0.5 % yeast extract, 1 % NaCl, adjustment to pH 9 |
| TSB | 3 % trypticase soy broth |
| TSB pH 5 | 3 % trypticase soy broth, adjustment to pH 5 |
| TSB pH 9 | 3 % trypticase soy broth, adjustment to pH 9 |
| M9 | 12.8 g Na_2_HPO_4_.7H_2_O, 3 g KH_2_PO_4_, 0.5 g NaCl, 1 g NH_4_Cl (for 1 liter). 0.4 % glucose, 2 mM MgSO4, 0.1 mM CaCl_2_ are added after autoclaving |
| King B | 20 g proteose peptone #2, 1.5 g K_2_HPO_4_, 10 ml glycerol, 1.5 g MgSO_4_.7H_2_O (for 1 liter) |
| Rhizosphere soil medium | 3 mM glucose, 10 % soil extract (maize, bean or garden compost) (Yoder-Himes et al. 2009) |

**Table S3.** List of primers used in this study.

| **Primer number** | **Primer sequence** | **Purpose of use** |
| --- | --- | --- |
| PGPRB-5104 | TGGCAGCAGCCAACTCAGCTT | Reverse sequencing primer pET28a |
| PGPRB-5105 | TATAGGCGCCAGCAACCGCA | Forward sequencing primer pET28a |
| PGPRB-6368 | TTGCTA**CATATG**AAACGACTTGAGCCGGGC | Forward primer used to construct pCMPG6200 (cloning of Bamb_0926 from *B. ambifaria* MEX5) |
| PGPRB-6371 | TTGCTA**CTCGAG**TCAGAACGGAAGTGAAATGATCGC | Reverse primer used to construct pCMPG6200 (cloning of Bamb_0926 from *B. ambifaria* MEX5) |
| PGPRB-6373 | TTGCTA**CATATG**AAGATCCTTGGCTCAAATGAAACG | Forward primer used to construct pCMPG6192 (cloning of Bcen_1091 from *B. cenocepacia* AU1054) |
| PGPRB-6376 | TTGCTA**CTCGAG**TCAGAAGGCGGCACCC | Reverse primer used to construct pCMPG6192 (cloning of Bcen_1091 from *B. cenocepacia* AU1054) |
| PGPRB-6378 | TTGCTA**CATATG**ACGGTGATTCCTTCTGGGG | Forward primer used to construct pCMPG6196 (cloning of Bcen_1092 from *B. cenocepacia* AU1054) |
| PGPRB-6381 | TTGCTA**CTCGAG**TTAGAACGGAATTGAGATTGGCTTC | Reverse primer used to construct pCMPG6196 (cloning of Bcen_1092 from *B. cenocepacia* AU1054) |

The restriction sites incorporated in the primers are in bold: CATATG, NdeI; CTCGAG, XhoI.

**Figure S1.** Multiple amino acid sequence alignment used to construct the phylogenetic tree of LlpA homologues (Fig. 1). The protein codes and accession numbers are specified in Fig. 1. Sequence conservation is visualized by differential shading. The position of the sequences corresponding to QxDxNxVxY-like motifs of MMBL proteins, representing potential carbohydrate-binding pockets, and the separate domains (N- and C-domain with MMBL fold; β-hairpin extension) present in *P. putida* BW11M1 LlpA (PDB 3M7H) are indicated.


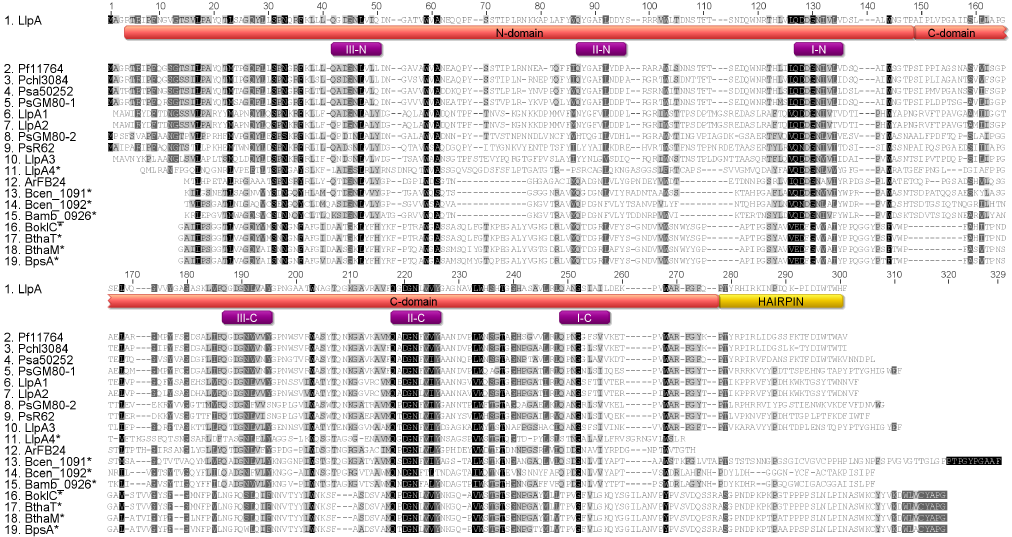


**Figure S2.** Glycan array profile of LlpA (Bcen_1091 from *B. cenocepacia* AU1054) as measured by fluorescence intensity. A complete list of tested carbohydrates (array version PA v5) is available from the Consortium of Functional Glycomics (CFG, www.functionalglycomics.org).

**References**

1. De Mot, R., Schoofs, G., Roelandt, A., Declerck, P., Proost, P., Van Damme, J., and J. Vanderleyden. 1994. Molecular characterization of the major outer-membrane protein OprF from plant root-colonizing *Pseudomonas fluorescens*. Microbiology 140(Pt6):1377-1387
2. Franklin, F. C., Bagdasarian, M., Bagdasarian, M. M., and K. N. Timmis. 1981. Molecular and functional analysis of the TOL plasmid pWWO from *Pseudomonas putida* and cloning of genes for the entire regulated aromatic ring meta cleavage pathway. Proc. Natl. Acad. Sci. U S A 78(12):7458-7462.
3. Rainey, P. B., and M. J. Bailey. 1996. Physical and genetic map of the *Pseudomonas fluorescens* SBW25 genome. Mol. Microbiol. 19(3):521-533
4. Shanahan, P., O’Sullivan, D. J., Simpson, P., Glennon, J. D., and F. O’Gara. 1992. Isolation of 2,4-diacetylphloroglucinol from a fluorescent pseudomonad and investigation of physiological parameters influencing its production. Appl. Environ. Microbiol. 58(1):353-358
5. Sebaihia, M., Preston, A., Maskell, D. J., Kuzmiak, H., Connell, T. D., King, N. D., Orndorff, P. E., Miyamoto, D. M., Thomson, N. R., Harris, D., Goble, A., Lord, A., Murphy, L., Quail, M. A., Rutter, S., Squares, R., Squares, S., Woodward, J., Parkhill, J., and L. M. Temple. 2006. Comparison of the genome sequence of the poultry pathogen *Bordetella avium* with those of *B. bronchiseptica*, *B. pertussis*, and *B. parapertussis* reveals extensive diversity in surface structures associated with host interaction. J. Bacteriol. 188(16):6002-6015.
6. Throup, J., Winson, M. K., Bainton, N. J., Bycroft, B. W., Williams, P., and G. S. A. B. Stewart. 1995. Signalling in bacteria beyond bioluminescence. In: Campbell, A., Kricka, L., and P. Stanley (eds) Bioluminscence and Chemiluminescence: Fundamentals and Applied Aspects. Chichester (UK): Wiley & Sons, p. 89-92
7. Vlassak, K., Van Holm, L., Duchateau, L., Vanderleyden, J., and R. De Mot. 1992. Isolation and characterization of fluorescent *Pseudomonas* associated with the roots of rice and banana grown in Sri Lanka. Plant Soil 145(1):51-63
8. Weller, D. M., and R. J. Cook. 1983. Suppression of take-all of wheat by seed treatments with fluorescent pseudomonads. Phytopathology 73(3):463-469
9. Yoder-Himes, D. R., Chain, P. S., Zhu, Y., Wurtzel, O., Rubin, E. M., Tiedje, J. M., and R. Sorek. 2009. Mapping the *Burkholderia cenocepacia* niche response via high-throughput sequencing. Proc. Natl. Acad. Sci. U S A 106(10):3976-3981
